# Implementation of an educational intervention to improve medical student cost awareness: a prospective cohort study

**DOI:** 10.1186/s12909-023-04038-1

**Published:** 2023-01-30

**Authors:** Sarah D Tait, Sachiko M Oshima, Harold J Leeras, Alexander Gunn, Melissa Sarver, Funda Gunes, Rachel A. Greenup

**Affiliations:** 1 Department of Internal Medicine, Massachusetts General Hospital, MA Boston, USA; 2 Department of Internal Medicine, Duke University School of Medicine, NC Durham, USA; 3 Department of Surgery, Duke University School of Medicine, NC Durham, USA; 4 Duke University School of Medicine, NC Durham, USA; 5 Department of Statistical Science, Duke University, NC Durham, USA; 6 Department of Surgery, Yale University, 310 Cedar Street, LH 118, 60510 New Haven, Connecticut, USA

**Keywords:** Financial toxicity, Financial hardship, Medical education, High-value care

## Abstract

**Background:**

In the context of rising healthcare costs, formal education on treatment-related financial hardship is lacking in many medical schools, leaving future physicians undereducated and unprepared to engage in high-value care.

**Method:**

We performed a prospective cohort study to characterize medical student knowledge regarding treatment-related financial hardship from 2019 to 2020 and 2020–2021, with the latter cohort receiving a targeted educational intervention to increase cost awareness. Using Kirkpatrick’s four-level training evaluation model, survey data was analyzed to characterize the acceptability of the intervention and the impact of the intervention on student knowledge, attitudes, and self-reported preparedness to engage in cost-conscious care.

**Results:**

Overall, *N* = 142 medical students completed the study survey; 61 (47.3%) in the non-intervention arm and 81 (66.4%) in the intervention arm. Of the 81 who completed the baseline survey in the intervention arm, 65 (80.2%) completed the immediate post-intervention survey and 39 (48.1%) completed the two-month post-intervention survey. Following the educational intervention, students reported a significantly increased understanding of common financial terms, access to cost-related resources, and level of comfort and preparedness in engaging in discussions around cost compared to their pre-intervention responses. The majority of participants (97.4%) reported that they would recommend the intervention to future students. A greater proportion of financially stressed students reported considering patient costs when making treatment decisions compared to their non-financially stressed peers.

**Conclusions:**

Targeted educational interventions to increase cost awareness have the potential to improve both medical student knowledge and preparedness to engage in cost-conscious care. Student financial stress may impact high-value care practices. Robust curricula on high-value care, including treatment-related financial hardship, should be formalized and universal within medical school training.

**Supplementary Information:**

The online version contains supplementary material available at 10.1186/s12909-023-04038-1.

## Background

Over the past several decades, healthcare costs have grown exponentially, with recent estimates citing healthcare spending as 18% of total economic expenditures [1]. Cost-sharing has resulted in patients shouldering a greater proportion of the financial burden related to medical care [2–4]. Treatment-related financial hardship is best described within cancer care due to an aging patient population and advances in costly therapies; however, a similar phenomenon has been observed in other areas of medical care, including among individuals with chronic health conditions [5–9]. Significant to catastrophic financial burden is associated with increased patient mortality and treatment non-adherence, as well as a notable decrease in quality of life [10–16]. There are several terms used to describe the phenomenon of treatment-related financial hardship, with financial burden describing the quantified cost, financial distress describing the perceived effect of the cost on the patient, and financial toxicity describing the associated patient outcomes.

Treatment-related financial hardship is gaining recognition on a national level and is the subject of ongoing research [17–19]. Multiple patient-facing interventions have been proposed to address this problem, including bedside cost communication and financial navigation, with efforts underway to increase physician awareness and incentivize high-value care. Although physicians report recognizing the impact of high healthcare costs on their patients, they also consistently report feeling ill-prepared for cost discussions and express a perceived inability to help [20, 21]. This gap in physician preparedness to address treatment-related financial hardship begins early on in training. Education to promote cost awareness has yet to be formalized in many medical school curricula, and a significant number of medical students report that they lack the skills to apply the principles of high-value care in medical decision-making, leaving students unequipped to engage in cost discussions with their patients and to consider the financial repercussions of treatment decisions as they transition into roles as healthcare providers [22, 23]. Preliminary studies have shown that educational initiatives targeted towards medical students can be effective in promoting cost awareness, and the Association of American Medical Colleges (AAMC) now includes implementation of cost-effective principles as part of its recommended Core Entrustable Professional Activities for medical students [24, 25]. Thus, we sought to pilot a targeted educational intervention aimed at improving cost awareness among medical students. While research is ongoing regarding how to best assess medical competence and advances in medical education, we utilized self-assessments to evaluate the impact of our intervention, which are a common method of assessment for student knowledge and behaviors [26].

## Methods

### Study design and participants

**Fig. 1:**
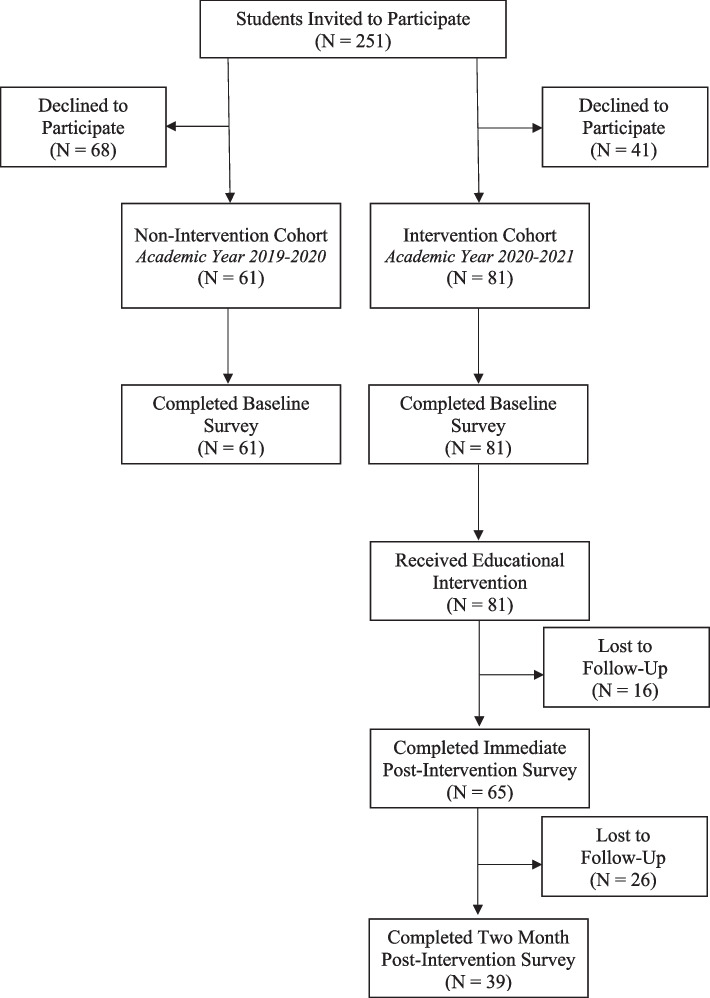
Participant Flow Diagram: Medical Student Participants in Academic Years 2019–2020 and 2020–2021

We performed a prospective cohort study using survey data collected from medical students during their clinical year of training including the academic years of 2019–2020 and 2020–2021. The study consisted of two arms: (i) the non-intervention arm (2019–2020); and, (ii) the intervention arm (2020–2021). Study schema is outline in Fig. 1 and study timeline is outlined in Additional File 1. All participants completed the baseline survey assessing cost awareness (Additional File 2). Medical students in the intervention arm received a targeted educational intervention as part of their clinical skills curriculum and completed two additional surveys, one immediately following the intervention and one 2 months after the intervention, assessing the acceptability of the intervention and its impact on student knowledge, attitude, and preparedness to engage in cost discussions (Additional files 3 and 4). Participants who completed all relevant study surveys were entered into a lottery for a $100 gift card. This study received approval from our Institutional Review Board.

### Educational intervention

The targeted educational intervention included: (a) formal didactic lecture; (b) 60-minute small group exercise; and, (c) a pocket card that was electronically distributed. The didactic lecture included an introduction to the concept of treatment-related financial hardship. It concluded with an exploration of opportunities to incorporate cost-efficiency and high-value care into medical decision-making. The small group exercise involved case-based discussion of patient out-of-pocket costs, insurance coverage, cost-effective diagnostics, cost-conscious.

treatment considerations, and differences in outpatient, inpatient, and emergency department charges. Lastly, the pocket card included a screening tool to identify patients at risk for treatment-related financial hardship, [27] definitions of common out-of-pocket costs, and region-specific resources to offer patients suffering from treatment-related financial hardship.

### Survey design

Medical students in both arms received an electronic link to an anonymous baseline survey in REDCap, a secure online database, which took approximately 5 min to complete. The invitation to participate included a brief overview of the concept of treatment-related financial hardship and the need for further research regarding physician cost awareness. Both post-intervention surveys were similarly distributed to the intervention cohort, with survey questions including validated, Likert-scale measures from previously published literature [20, 27, 28]. Survey items assessed participant sociodemographic factors, self-reported financial security, prior exposure to cost-related educational activities, acceptability of the educational intervention, and engagement in both cost awareness and cost communication with patients and medical teams.

### Outcome measures

Primary outcome measures were based upon Kirkpatrick’s four-level training evaluation model, [29] and included: acceptability of the educational intervention; student knowledge regarding treatment-related financial hardship; student attitudes regarding cost-conscious practices; and student self-reported comfort and preparedness to engage in cost communication with patients and medical teams as proxies for student behavior.

### Statistical analysis

Descriptive statistics were used to summarize participant characteristics and survey responses in both cohorts. Continuous and categorical variables were summarized with median (interquartile range, IQR) and N (%), respectively. For the intervention cohort, differences in the primary outcome measures pre- and post-intervention were tested using the McNemar test. Pre-intervention differences in cost awareness knowledge and practices were compared by reported level of financial stress using the Chi square test. Respondents who indicated that they were “always,” “very often,” or “sometimes” financially stressed were classified as “financially stressed”, whereas respondents who indicated that they were “rarely” or “never” financially stressed were classified as “not financially stressed”. For all analyses, the threshold for significance was set at level α < 0.05. All statistical analyses were conducted with SAS version 9.4 (SAS Institute, Cary NC).

## Results

### Demographic characteristics

The overall study cohort included *N* = 142 participants. Of the students invited to participate in the baseline knowledge assessment survey, 47.3% (61/129) responded in the non-intervention arm and 66.4% (81/122) in the intervention arm. Enrollment details are outlined in Fig. 1. Participants in the intervention arm received an immediate post-intervention survey, of which 80.2% (65/81) responded, as well as a two-month post-intervention survey, of which 48.1% responded (39/81). In addition, the cohort exposed to the educational intervention completed an evaluation of its acceptability with a response rate of 95% (116/122). Baseline characteristics of both cohorts are presented in Table 1.

**Table 1:** Baseline characteristics of the non-intervention and intervention cohorts, academic years 2019–2020 and 2020–2021

	Non-Intervention Cohort *N* = 61 *Median (IQR) or* *N (%)*	Intervention Cohort *N* = 81 *Median (IQR) or* *N (%)*
Age (Years)		
Median (IQR)	25 (25–26)	24 (24–25)
Race/Ethnicity		
Non-Hispanic Asian	20 (32.8%)	20 (24.7%)
Hispanic	8 (13.1%)	10 (12.4%)
Non-Hispanic Black	2 (3.3%)	4 (4.9%)
Non-Hispanic White	28 (45.9%)	34 (42.0%)
Other, More than one, or Prefer not to answer	3 (4.9%)	13 (16.1%)
Gender		
Male	21 (34.4%)	18 (22.2%)
Female	40 (65.6%)	59 (72.8%)
Other or Prefer not to answer	0 (0%)	4 (4.9%)
Relationship Status		
Single	25 (41.0%)	44 (54.3%)
Partnered	34 (42.0%)	34 (42.0%)
Other or Prefer not to answer	2 (3.3%)	3 (3.7%)
How frequently financially stressed		
Always, Very often, or Sometimes	47 (77.1%)	53 (65.4%)
Rarely or Never	14 (23.0%)	28 (34.6%)
Familiar with concept of financial toxicity?		
Yes	36 (59.0%)	34 (58.0%)
No	25 (41.0%)	47 (42.0%)
Received prior lecture on topic of financial toxicity?		
Yes	19 (31.2%)	16 (19.8%)
No	42 (68.9%)	65 (80.3%)

In the non-intervention cohort, the median age was 25 years (IQR 25–26) and 65.6% of participants were female. The majority (77.1%, *n* = 47) of respondents in the non-intervention cohort reported feeling financially stressed always, very often, or sometimes. In the intervention cohort, the median age was 24 years (IQR 24–25) and most respondents were female (72.8%, *n* = 59). The majority of participants (65.4%, *n* = 53) reported feeling financially stressed always, very often, or sometimes.

### Cost awareness knowledge, attitudes, and behaviors in the non-intervention cohort

In the non-intervention cohort, 51.7% (*n* = 31) of respondents agreed or strongly agreed that they had a good understanding of common financial terms (i.e., deductible, co-payment, co-insurance, and maximum out-of-pocket cost) (Table 2). Only 8.5% (*n* = 5) of respondents agreed or strongly agreed that they had easy access to quality resources to assist in cost discussions with patients. Most participants (75.4%, *n* = 46) agreed or strongly agreed that doctors should explain the cost of treatments to patients and 85.3% (*n* = 52) agreed or strongly agreed that doctors should consider costs when making treatment decisions for patients. When asked to reflect on their own practices, 67.2% (*n* = 41) reported that they considered patient out-of-pocket costs sometimes, most of the time, or all the time when making treatment decisions. However, only 9.8% (*n* = 6) of respondents agreed or strongly agreed that they felt prepared to discuss costs of treatment with patients, while only 21.3% (*n* = 13) agreed or strongly agreed that they felt comfortable discussing costs with patients.

**Table 2:** Cost awareness knowledge, attitudes, and behaviors in non-intervention cohort, academic year 2019–2020

	Non-Intervention Cohort *N* = 61 *Median (IQR) or* *N (%)*
“I have a good understanding of the following terms: deductibles, co-payment, co-insurance, maximum out of pocket cost”	
Strongly agree or Agree	31 (51.7%)
Neither agree nor disagree, Disagree, or Strongly disagree	29 (48.3%)
Frequency of considering patient out-of-pocket costs when making treatment decisions	
Never or Infrequently	20 (32.8%)
Sometimes, Most of the time, or All the time	41 (67.2%)
“When choosing treatment, doctors should consider costs to the patient”	
Strongly agree or Agree	52 (85.3%)
Neither agree nor disagree, Disagree, or Strongly disagree	9 (14.8%)
“I feel prepared to discuss costs of treatment with patients”	
Strongly agree or Agree	6 (9.8%)
Neither agree nor disagree, Disagree, or Strongly disagree	55 (90.2%)
“I feel comfortable discussing costs of treatment with patients”	
Strongly agree or Agree	13 (21.3%)
Neither agree nor disagree, Disagree, or Strongly disagree	48 (78.7%)
Frequency of engaging in cost discussions with patients	
Never or Infrequently	41 (67.2%)
Sometimes, Most of the time, or All the time	20 (32.8%)
Frequency of engaging in cost discussions with medical teams	
Never or Infrequently	22 (36.1%)
Sometimes, Most of the time, or All the time	39 (63.9%)
“I have easy access to quality resources that assist me in cost discussions with patients”	
Strongly agree or Agree	5 (8.5%)
Neither agree nor disagree, Disagree, or Strongly disagree	54 (91.5%)

### Cost awareness knowledge, attitudes, and behaviors in the intervention cohort

Significant differences were observed in participant responses in the intervention cohort pre- vs. post-intervention (Table 3). While 54.4% (*n* = 43) of respondents agreed or strongly agreed that they had a good understanding of common financial terms pre-intervention, 71.9% (*n* = 46) of respondents agreed or strongly agreed with this statement post-intervention (*p* < .001), with 23.8% (*n* = 15) of respondents who answered both surveys changing their response from a neutral or negative response to a positive response. Similarly, there was a significant increase in the proportion of participants who agreed or strongly agreed that they had access to quality resources to assist in cost discussions pre- vs. post-intervention (3.8% vs. 39.1%; *p* < .001), with 36.5% (*n* = 23) of students changing their response from neutral or negative to positive. Only 18.8% (*n* = 15) of respondents agreed or strongly agreed with the statement, “I feel comfortable discussing costs of treatment with patients” pre-intervention, while 46.9% (*n* = 30) respondents agreed or strongly agreed with this statement post-intervention (*p* < .001), with 28.6% (*n* = 18) of respondents changing their response from neutral or negative to positive post-intervention. Finally, a similar trend was observed in the proportion of respondents who agreed or strongly agreed that they felt prepared to discuss treatment costs with patients pre- vs. post-intervention (2.5% vs. 26.6%; *p* < .001), with 25.4% (*n* = 16) students changing their response from negative or neutral to positive following the intervention. There was no significant difference in the proportion of respondents who agreed or strongly agreed that doctors should consider costs to the patient when making treatment decisions pre- vs. post-intervention (94.9% vs. 95.3%; *p* = .66). An analysis of a subset of participants (*n* = 39) at two-months following the intervention demonstrated persistent increases in self-reported access to resources, comfort, and preparedness to engage in cost discussions with patients as compared to pre-intervention but revealed no other significant findings in cost-conscious behavior changes.

**Table 3:** Cost awareness knowledge, attitudes, and behaviors in intervention cohort pre- vs. post-intervention, academic year 2020–2021

	Pre-Intervention *N* = 81 *Median (IQR) or* *N (%)*	Post-Intervention *N* = 65 *Median (IQR) or* *N (%)*	*P*-Value
“I have a good understanding of the following terms: deductibles, co-payment, co-insurance, maximum out of pocket cost”			0.01
Strongly agree or Agree	43 (54.4%)	46 (71.9%)	
Neither agree nor disagree, Disagree, or Strongly disagree	36 (45.6%)	18 (28.1%)	
“When choosing treatment, doctors should consider costs to the patient”			0.66
Strongly agree or Agree	75 (94.9%)	61 (95.3%)	
Neither agree nor disagree, Disagree, or Strongly disagree	4 (5.1%)	3 (4.7%)	
“I feel prepared to discuss costs of treatment with patients”			< 0.001
Strongly agree or Agree	2 (2.5%)	17 (26.6%)	
Neither agree nor disagree, Disagree, or Strongly disagree	78 (97.5%)	47 (73.4%)	
“I feel comfortable discussing costs of treatment with patients”			< 0.001
Strongly agree or Agree	15 (18.8%)	30 (46.9%)	
Neither agree nor disagree, Disagree, or Strongly disagree	65 (81.3%)	34 (53.1%)	
“I have easy access to quality resources that assist me in cost discussions with patients”			< 0.001
Strongly agree or Agree	3 (3.8%)	25 (39.1%)	
Neither agree nor disagree, Disagree, or Strongly disagree	77 (96.3%)	39 (60.9%)	

### Cost awareness behaviors by financial stress

In the intervention cohort, 65.5% (*n* = 53) of respondents reported feeling financially stressed. When cost awareness practices were compared by level of reported financial stress, respondents who were financially stressed were significantly more likely to consider patient out-of-pocket costs when making treatment decisions, with 81.1% of financially stressed respondents reporting they considered patient costs sometimes, most of the time, or all of the time as compared to 57.1% of non-financially stressed respondents; *p* = .02 (Table 4). Differences in the frequency with which respondents reported engaging in cost discussions with patients or medical teams based on level of financial stress were not significant.

**Table 4:** Cost awareness knowledge, attitudes, and behaviors by medical student financial stress in intervention cohort pre-intervention, academic year 2020–2021

	Financially Stressed *N* = 53 *Median (IQR) or* *N (%)*	Not Financially Stressed *N* = 28 *Median (IQR) or* *N (%)*	*P*-Value
“I have a good understanding of the following terms: deductibles, co-payment, co-insurance, maximum out of pocket cost”			0.74
Strongly agree or Agree	29 (44.2%)	14 (51.9%)	
Neither agree nor disagree, Disagree, or Strongly disagree	23 (55.8%)	13 (48.2%)	
“When choosing treatment, doctors should consider costs to the patient”			0.69
Strongly agree or Agree	49 (94.2%)	26 (96.3%)	
Neither agree nor disagree, Disagree, or Strongly disagree	3 (5.8%)	1 (3.7%)	
“I feel prepared to discuss costs of treatment with patients”			0.62
Strongly agree or Agree	1 (1.9%)	1(3.7%)	
Neither agree nor disagree, Disagree, or Strongly disagree	52 (98.1%)	26 (96.3%)	
“I feel comfortable discussing costs of treatment with patients”			0.97
Strongly agree or Agree	10 (18.9%)	5 (18.5%)	
Neither agree nor disagree, Disagree, or Strongly disagree	43 (81.1%)	22 (81.5%)	
“I have easy access to quality resources that assist me in cost discussions with patients”			0.22
Strongly agree or Agree	1 (1.9%)	2 (7.4%)	
Neither agree nor disagree, Disagree, or Strongly disagree	52 (98.1%)	25 (92.6%)	
Frequency of considering patient out-of-pocket costs when making treatment decisions			0.02
Never or Infrequently	10 (18.9%)	12 (42.9%)	
Sometimes, Most of the time, or All the time	43 (81.1%)	16 (57.1%)	
Frequency of engaging in cost discussions with patients			0.17
Never or Infrequently	36 (67.9%)	23 (82.1%)	
Sometimes, Most of the time, or All the time	17 (32.1%)	5 (17.9%)	
Frequency of engaging in cost discussions with medical teams			0.32
Never or Infrequently	26 (49.1%)	17 (60.7%)	
Sometimes, Most of the time, or All the time	27 (50.9%)	11 (39.3%)	

### Acceptability of the educational intervention

The majority of students surveyed (97.4%, *n* = 113) reported that they would recommend the cost awareness lecture to future classes. Most participants (89.2%) indicated that they did not use the mobile cost awareness pocket tool after it was distributed.

## Discussion

Exponential rises in contemporary healthcare costs have resulted in significant monetary burden for individuals receiving medical care. In our survey study of medical students (*N* = 142) in their first clinical year of training, we found their familiarity with concepts related to cost awareness is lacking, with only half of students reporting a good understanding of key financial terms and less than 10% reporting easy access to resources to assist in cost discussions with patients. Not surprisingly, this lack of familiarity translates into lack of engagement with cost-conscious care. Less than 10% of respondents felt prepared to engage in cost discussions with patients, less than 30% felt comfortable engaging in such discussions, and about two-thirds reported never or infrequently participating in these discussions. Formal education regarding treatment-related financial hardship holds the potential to activate generations of future physicians to engage in cost-conscious care and communication. At the bedside, patient-provider cost communication posits to destigmatize inquiry about the affordability of care. At the national level, patient-provider cost communication engages physician leadership in policy-relevant discussions.

In the setting of growing patient financial burden, increased financial distress, and associated delays in seeking care, physicians have a responsibility to understand the financial implications of their treatment decisions and to work towards mitigating unaffordable costs that may serve as barriers to care [2, 3, 30, 31]. In order to prepare physicians to take on this role, medical training must involve formalized education in and exposure to health services delivery and high-value care [32]. Indeed, both the AAMC and the Accreditation Council for Graduate Medical Education (ACGME) now include implementation of cost-effective principles as a core competency alongside other fundamental clinical skills [24, 33]. Undergraduate medical education represents a unique time in medical training when trainees have both the time and resources to develop and practice principles of cost-conscious care. More importantly, this is a time in a young physician’s career when clinical habits are formed. Inquiry regarding financial security could and should be embedded into the routine social history, limiting provider bias around which patients may or may not be able to pay for healthcare. Despite this, most medical schools lack formalized curricula around affordability or high-value care [22].

Our findings suggest that medical student financial stress may impact baseline cost awareness. In our study, financially stressed respondents were more likely to report considering patient costs when making treatment recommendations. While financial stress has been shown to influence specialty choice and academic performance, this is the first study of its kind to demonstrate a potential impact on patient care practices among medical students [34]. Furthermore, existing evidence suggests that increased socioeconomic diversity of the physician workforce improves the quality of patient care; our findings may reflect that diversity among clinical trainees and faculty is associated with greater cost conscicousness and advocacy for patients [35]. As financial burden disproportionately impacts minority patient populations, ensuring diversity in trainee socioeconomic backgrounds may ultimately help to mitigate outcomes disparities [6, 36]. Unfortunately, a significant socioeconomic diversity gap persists in medical education, perpetuated both by substantial increases in the costs of medical school attendance and inherent difficulties in assessing socioeconomic background as a less visible form of diversity in the applications process [37–39]. Further work is needed to better understand the impact of provider financial stress on patient-provider interaction and patient outcomes, both health- and cost-related.

Importantly, our targeted educational intervention was effective in increasing medical student cost awareness and preparedness to engage in cost-conscious care, as evidenced by the significant increase that was observed in medical student understanding of key financial terms, reported access to cost-related resources, and self-reported level of comfort and preparedness to engage in cost discussions with patients. The educational intervention was shown to be acceptable to the vast majority of students, with 97% of respondents reporting they would recommend the experience to future students.

Our study is not the first to demonstrate that a targeted educational intervention to increase medical student cost awareness is both feasible and effective. Previous studies examining the implementation of cost-conscious educational curricula have also shown that these interventions can be effective in promoting medical student engagement in high-value care, [40–42] supporting the idea that curricula such as these should be universal in medical student training. Our study is the first, however, to assess students’ perceived obligation to consider costs when making treatment decisions. When surveyed after the educational intervention, over 95% of students affirmed physician responsibility to consider patient costs when making decisions. This compares to a national survey of over 2,500 practicing physicians where only 36% of respondents reported physicians have a “major responsibility” to reduce healthcare costs [43]. Clearly, there is no universal consensus amongst clinicians regarding the physician role in addressing healthcare costs and the heterogeneity in perception has the potential to create clinical learning environments in which trainees are disincentivized to practice and implement cost-conscious care. Encouragingly, educational interventions targeted towards medical students have been shown to increase engagement in high-value care not only for the students themselves but also for other clinical team members, suggesting a wide-ranging impact from interventions such as the one described here [40].

Future work is needed to explore the way in which prior exposure to the concept of treatment-related financial hardship impacts efficacy and acceptability of this educational intervention, how educational interventions such as these can alter institutional clinical culture, and to assess the longitudinal impacts of these curricula on medical student clinical behaviors during the transition to residency and beyond through multimodal evaluation. Additionally, though the overall student reception of the educational intervention was quite positive, most participants did not use the mobile pocket tool that was developed and distributed as part of the intervention, highlighting the ongoing need for the development of user-friendly resources to aid in cost discussions with patients that are both high-yield and practical to incorporate into routine clinical use.

Our study included several limitations that must be acknowledged. When averaged amongst both cohorts, the response rate for the baseline survey was 57%. Although higher response rates approximating 65% are typically desirable, our response rate is similar to or higher than those reported in prior survey studies of medical student cohorts [44, 45]. Additionally, a significant proportion of participants were lost to follow-up and did not complete the immediate or two-month post-intervention surveys, thus our findings may be subject to non-response bias and may not capture the true impact of the intervention on student knowledge and perception. Furthermore, our two-month post-intervention response rate limited our ability to assess the long-term behavioral and knowledge impacts of our intervention. Lastly, our exploratory findings are based on a small sample size from a single medical school associated with a quaternary care academic medical center and thus may not be generalizable to the greater medical student population. Future work focused on implementing and evaluating this curriculum at multiple sites is needed to more completely assess feasibility and efficacy.

## Conclusion

Our study demonstrated that a targeted educational intervention on treatment-related financial hardship and cost awareness has the potential to change both medical student knowledge and preparedness to engage in cost-conscious care. Additionally, we found that medical student financial stress may impact high-value care practices. Robust curricula on high-value care, including treatment-related financial hardship, should be formalized and universal within medical school training.

### Supplementary Information

**Additional file 1.** Study Timeline.

**Additional file 2.** Baseline Survey.

**Additional file 3.** Intervention Cohort Immediate Post-Session Follow-Up Survey.

**Additional file 4.** Intervention Cohort Two Month Post-Session Follow-Up Survey

## Data Availability

The datasets generated and analysed during this study are available from the corresponding author on reasonable request.

## References

[1] Hartman M, Martin AB, Benson J, Catlin A, National Health Expenditure Accounts T. National Health Care Spending In 2018 (2020). Growth Driven by Accelerations in Medicare and private insurance spending. Health Aff (Millwood).

[2] 2019 Employer Health Benefits Survey: Henry J. Kaiser Family Foundation. ; 2019 Available from: https://www.kff.org/report-section/ehbs-2019-summary-of-findings/.

[3] Banthin JS, Bernard DM (2006). Changes in financial burdens for health care: national estimates for the population younger than 65 years, 1996 to 2003. JAMA.

[4] Cunningham PJ (2010). The growing financial burden of health care: national and state trends, 2001–2006. Health Aff (Millwood).

[5] Elkin EB, Bach PB (2010). Cancer’s next frontier: addressing high and increasing costs. JAMA.

[6] Abdus S, Keenan PS (2018). Financial Burden of Employer-Sponsored High-Deductible Health Plans for low-income adults with Chronic Health Conditions. JAMA Intern Med.

[7] Yabroff KR, Lund J, Kepka D, Mariotto A (2011). Economic burden of cancer in the United States: estimates, projections, and future research. Cancer Epidemiol Biomarkers Prev.

[8] Ramsey S, Blough D, Kirchhoff A, Kreizenbeck K, Fedorenko C, Snell K (2013). Washington State Cancer Patients found to be at Greater Risk for Bankruptcy Than People without a Cancer diagnosis. Health Affair.

[9] Slavin SD, Khera R, Zafar SY, Nasir K, Warraich HJ (2021). Financial burden, distress, and toxicity in cardiovascular disease. Am Heart J.

[10] Pisu M, Kenzik KM, Oster RA, Drentea P, Ashing KT, Fouad M (2015). Economic hardship of minority and non-minority cancer survivors 1 year after diagnosis: another long-term effect of cancer?. Cancer.

[11] Kent EE, Forsythe LP, Yabroff KR, Weaver KE, de Moor JS, Rodriguez JL (2013). Are survivors who report cancer-related financial problems more likely to forgo or delay medical care?. Cancer.

[12] Ramsey SD, Bansal A, Fedorenko CR, Blough DK, Overstreet KA, Shankaran V (2016). Financial Insolvency as a risk factor for early mortality among patients with Cancer. J Clin Oncol.

[13] Wharam JF, Zhang F, Lu CY, Wagner AK, Nekhlyudov L, Earle CC (2018). Breast Cancer diagnosis and treatment after high-deductible insurance enrollment. J Clin Oncol.

[14] Okunrintemi V, Valero-Elizondo J, Michos ED, Salami JA, Ogunmoroti O, Osondu C (2019). Association of Depression Risk with patient experience, Healthcare Expenditure, and Health Resource utilization among adults with atherosclerotic Cardiovascular Disease. J Gen Intern Med.

[15] Ngo-Metzger Q, Sorkin DH, Billimek J, Greenfield S, Kaplan SH (2012). The effects of financial pressures on adherence and glucose control among racial/ethnically diverse patients with diabetes. J Gen Intern Med.

[16] Dodd R, Palagyi A, Guild L, Jha V, Jan S (2018). The impact of out-of-pocket costs on treatment commencement and adherence in chronic kidney disease: a systematic review. Health Policy Plan.

[17] Tucker-Seeley RD, Yabroff KR. Minimizing the “Financial Toxicity” Associated With Cancer Care: Advancing the Research Agenda. J Natl Cancer Inst. 2015;108(5):djv410. 10.1093/jnci/djv410 26657336

[18] de Souza JA, Yap B, Ratain MJ, Daugherty C (2015). User beware: we need more science and less art when measuring financial toxicity in oncology. J Clin Oncol.

[19] Meropol NJ, Schrag D, Smith TJ, Mulvey TM, Langdon RM, Blum D (2009). American Society of Clinical Oncology Guidance Statement: the cost of Cancer Care. J Clin Oncol.

[20] Greenup RA, Rushing CN, Fish LJ, Lane WO, Peppercorn JM, Bellavance E (2019). Perspectives on the costs of Cancer Care: a Survey of the american society of breast surgeons. Ann Surg Oncol.

[21] Altomare I, Irwin B, Zafar SY, Houck K, Maloney B, Greenup R (2016). Physician experience and attitudes toward addressing the cost of Cancer Care. J Oncol Pract.

[22] Cayea D, Tartaglia K, Pahwa A, Harrell H, Shaheen A, Lang VJ (2018). Current and optimal training in high-value care in the Internal Medicine Clerkship: A National Curricular needs Assessment. Acad Med.

[23] American Association of Medical Colleges. Medical school graduation questionnaire: 2020 all schools summary report. 2020.

[24] Obeso V, Brown D, Aiyer M, Barron B, Bull J, Carter T, et al. Toolkits for the 13 core entrustable professional activities for entering residency. Washington: Association of American Medical Colleges. 2017.

[25] Stammen LA, Stalmeijer RE, Paternotte E, Oudkerk Pool A, Driessen EW, Scheele F (2015). Training Physicians to provide High-Value, cost-conscious care: a systematic review. JAMA.

[26] Epstein RM (2007). Assessment in medical education. New Engl J Med.

[27] de Souza JA, Yap BJ, Wroblewski K, Blinder V, Araújo FS, Hlubocky FJ (2017). Measuring financial toxicity as a clinically relevant patient-reported outcome: the validation of the COmprehensive score for financial toxicity (COST). Cancer.

[28] Greenup RA, Rushing C, Fish L, Campbell BM, Tolnitch L, Hyslop T (2019). Financial costs and Burden related to decisions for breast Cancer surgery. J Oncol Pract.

[29] Bates R (2004). A critical analysis of evaluation practice: the Kirkpatrick model and the principle of beneficence. Eval Program Plan.

[30] Zafar SY, Newcomer LN, McCarthy J, Fuld Nasso S, Saltz LB (2017). How should we intervene on the Financial toxicity of Cancer Care? One shot, four perspectives. Am Soc Clin Oncol Educ Book.

[31] Emanuel EJ, Glickman A, Johnson D (2017). Measuring the Burden of Health Care costs on US families: the Affordability Index. JAMA.

[32] Cooke M (2010). Cost consciousness in patient care–what is medical education’s responsibility?. N Engl J Med.

[33] Accreditation Council for Graduate Medical Education. ACGME common program requirements (residency). Accreditation Council for Graduate Medical Education. 2022.

[34] Pisaniello MS, Asahina AT, Bacchi S, Wagner M, Perry SW, Wong ML (2019). Effect of medical student debt on mental health, academic performance and specialty choice: a systematic review. BMJ Open.

[35] Gomez LE, Bernet P (2019). Diversity improves performance and outcomes. J Natl Med Assoc.

[36] Huey RW, George GC, Phillips P, White R, Fu S, Janku F (2021). Patient-reported out-of-Pocket costs and financial toxicity during early-phase oncology clinical trials. Oncologist.

[37] Millo L, Ho N, Ubel PA (2019). The cost of applying to Medical School — a barrier to diversifying the Profession. N Engl J Med.

[38] Shahriar AA, Puram VV, Miller JM, Sagi V, Castañón-Gonzalez LA, Prasad S (2022). Socioeconomic diversity of the matriculating US Medical Student body by race, ethnicity, and sex, 2017–2019. JAMA Netw Open.

[39] Le HH (2017). The socioeconomic diversity gap in Medical Education. Acad Med.

[40] Lacy M, Noronha L (2020). High-value care for senior medical students. Clin Teach.

[41] Pahwa AK, Eaton K, Apfel A, Bertram A, Ridell R, Cayea D (2020). Effect of a high value care curriculum on standardized patient exam in the Core Clerkship in Internal Medicine. BMC Med Educ.

[42] Steele C, Cayea D, Berk J, Riddell R, Kumra T, McGuire M (2019). Novel first-year curriculum in high-value care. Clin Teach.

[43] Tilburt JC, Wynia MK, Sheeler RD, Thorsteinsdottir B, James KM, Egginton JS (2013). Views of US physicians about controlling health care costs. JAMA.

[44] Onello E, Friedrichsen S, Krafts K, Simmons G, Diebel K (2020). First year allopathic medical student attitudes about vaccination and vaccine hesitancy. Vaccine.

[45] Rogers AJG (2020). Medical student volunteerism and interest in working with underserved and vulnerable populations. BMC Med Educ.

