# Correction: Implementation of an educational intervention to improve medical student cost awareness: a prospective cohort study

**DOI:** 10.1186/s12909-023-04797-x

**Published:** 2023-10-25

**Authors:** Sarah D. Tait, Sachiko M. Oshima, Harold J. Leraas, Alexander Gunn, Melissa Sarver, Funda Gunes, Rachel A. Greenup

**Affiliations:** 1 Department of Internal Medicine, Massachusetts General Hospital, Boston, MA USA; 2 Department of Internal Medicine, Duke University School of Medicine, Durham, NC USA; 3 Department of Surgery, Duke University School of Medicine, Durham, NC USA; 4 Duke University School of Medicine, Durham, NC USA; 5 Department of Statistical Science, Duke University, Durham, NC USA; 6 Department of Surgery, Yale University, 310 Cedar Street, LH 118, New Haven, CT 60510 USA

**Correction: BMC Med Educ 23; 73 (2023)**

**https://doi.org/10.1186/s12909-023-04038-1**

Following publication of the original article [1], we have been informed that the name of the author Harold J Leraas has a spelling.

The incorrect name is: Harold J Leeras

The correct name is: Harold J Leraas
