# Supplementary material for: Implementation of an educational intervention to improve medical student cost awareness: a prospective cohort study
Source: BMC Med Educ. 2023 Jan 30;23:73. doi: 10.1186/s12909-023-04038-1 (PMC9885673; doi:10.1186/s12909-023-04038-1)
Supplement: Supplementary file 1 — Additional file 1. Study Timeline. [file 12909_2023_4038_MOESM1_ESM.docx]

**Additional File 1. Study Timeline**

**Educational Intervention to Improve Medical Student Cost Awareness: Study Timeline**

*Class of 2022 clinical year completed August 2020

**Class of 2023 clinical year started August 2020
