# Supplementary material for: Implementation of an educational intervention to improve medical student cost awareness: a prospective cohort study
Source: BMC Med Educ. 2023 Jan 30;23:73. doi: 10.1186/s12909-023-04038-1 (PMC9885673; doi:10.1186/s12909-023-04038-1)
Supplement: Supplementary file 2 — Additional file 2. Baseline Survey. [file 12909_2023_4038_MOESM2_ESM.docx]

**Additional File 2.** Baseline Survey

1. To maintain anonymity, please follow the instructions to create an ID that will be used to match your pre- and post-participation surveys.

-Initial of first name

-Last letter of last name

-First letter of birth month

-First letter of mother’s maiden name

Example: Susan Smith born in June, mother’s maiden name Jones: SHJJ

1. What is your age?
2. Which of the following describes your race or origin?
   1. White
   2. Black or African American
   3. Asian
   4. Native American or Alaska Native
   5. Native Hawaiian or other Pacific Islander
   6. Other
   7. More than one
   8. Prefer not to answer
3. What is your gender?
4. Male
5. Female
6. Other
7. Prefer not to answer
8. What is your relationship status?
9. Single
10. Partnered
11. Other
12. Prefer not to answer
13. Please indicate your response to the following statement: I feel financially stressed
14. Not at all
15. A little bit
16. Somewhat
17. Quite a bit
18. Very much
19. I am able to meet my monthly expenses:
20. Not at all
21. A little bit
22. Somewhat
23. Quite often
24. Very often
25. How possible would it be for you to come up with $2000 in the next month to deal with an emergency?
26. I’m not sure
27. Not at all possible
28. Not very possible
29. Somewhat possible
30. Very possible
31. Have you heard about the concept of financial toxicity before?
32. Yes
33. No

If yes, where? ___________

1. Have you received a lecture (i.e. through an extracurricular experience, outside of medical school, etc.) on financial toxicity before?
2. Yes
3. No

If yes, where? ___________

1. What is your knowledge level on topics like insurance systems, co-insurance, deductibles, and co-payments?
2. Very good
3. Good
4. Intermediate
5. Weak
6. In your experience, how often do patients consider their personal out-of-pocket or indirect costs when making treatment decisions?
7. All of the time
8. Most of the time
9. Sometimes
10. Infrequently
11. Never
12. How often do you think about patient out-of-pocket costs (i.e. deductibles and co-pays) when considering treatment options?
13. All of the time
14. Most of the time
15. Sometimes
16. Infrequently
17. Never
18. How often do you engage in cost discussions with patients?
19. All of the time
20. Most of the time
21. Sometimes
22. Infrequently
23. Never
24. Which of the following are barriers for you to discussing costs of treatment with patients? (select all that apply)
25. I don’t know enough about the costs of care/lack resources
26. Not enough time to discuss costs
27. I can’t help with the costs of care
28. It’s not my place to discuss costs of care
29. It is uncomfortable to discuss costs with patients
30. Discussing costs might impact the quality of care patients receive
31. Nothing prevents me from discussing costs
32. Other
33. How often do you engage in cost discussions with the rest of the medical team?
34. All of the time
35. Most of the time
36. Sometimes
37. Infrequently
38. Never
39. Which of the following are barriers for you to discussing costs of treatment with the rest of the medical team? (select all that apply)
40. I don’t know enough about the costs of care/lack resources
41. Not enough time to discuss costs
42. I can’t help with the costs of care
43. It’s not my place to discuss costs of care
44. It is uncomfortable to discuss costs with the medical team
45. Discussing costs might impact the quality of care patients receive
46. Nothing prevents me from discussing costs
47. Other
48. The following statements are aimed at helping us understand how health care providers think about treatment costs- including costs to patients and the health care system. Please indicate how much you agree or disagree.

|  | **Strongly**  **Agree** | **Agree** | **Neither Agree nor Disagree** | **Disagree** | **Strongly Disagree** |
| --- | --- | --- | --- | --- | --- |
| 1. I have a good understanding of the following terms: deductibles, co-payment, co-insurance, maximum out of pocket cost |  |  |  |  |  |
| 1. Doctors should explain to patients the costs the patient will have to pay for his or her treatment |  |  |  |  |  |
| 1. When choosing treatment, doctors should consider costs to the patient |  |  |  |  |  |
| 1. When choosing treatment, doctors should consider costs to society (i.e. how treatment of individual patients affects the health care system) |  |  |  |  |  |
| 1. Patients should have access to the costs of their treatment before making treatment decisions |  |  |  |  |  |
| 1. I feel prepared to discuss costs of treatment with patients |  |  |  |  |  |
| 1. I feel comfortable discussing costs of treatment with patients |  |  |  |  |  |
| 1. I have easy access to quality resources that assist me in cost discussions with patients |  |  |  |  |  |
| 1. My consideration of health care costs varies based on my patient’s insurance status or socioeconomic background. |  |  |  |  |  |
| 1. If two treatments are equally effective, I believe doctors should recommend the less expensive option |  |  |  |  |  |
