# Supplementary material for: Implementation of an educational intervention to improve medical student cost awareness: a prospective cohort study
Source: BMC Med Educ. 2023 Jan 30;23:73. doi: 10.1186/s12909-023-04038-1 (PMC9885673; doi:10.1186/s12909-023-04038-1)
Supplement: Supplementary file 3 — Additional file 3. Intervention Cohort Immediate Post-Session Follow-Up Survey. [file 12909_2023_4038_MOESM3_ESM.docx]

**Additional File 3.** Intervention Cohort Immediate Post-Session Follow-Up Survey

1. To maintain anonymity, please follow the instructions to create an ID that will be used to match your pre- and post-participation surveys.

-Initial of first name

-Last letter of last name

-First letter of birth month

-First letter of mother’s maiden name

Example: Susan Smith born in June, mother’s maiden name Jones: SHJJ

1. How helpful did you find this session on financial toxicity and high-value care?
2. Very helpful
3. Moderately helpful
4. Somewhat helpful
5. Not helpful
6. Would you recommend this session for future medical students?
   1. Yes
   2. No
7. The following statements are aimed at helping us understand how health care providers think about treatment costs- including costs to patients and the health care system. Please indicate how much you agree or disagree.

|  | **Strongly**  **Agree** | **Agree** | **Neither Agree nor Disagree** | **Disagree** | **Strongly Disagree** |
| --- | --- | --- | --- | --- | --- |
| 1. I have a good understanding of the following terms: deductibles, co-payment, co-insurance, maximum out of pocket cost |  |  |  |  |  |
| 1. Doctors should explain to patients the costs the patient will have to pay for his or her treatment |  |  |  |  |  |
| 1. When choosing treatment, doctors should consider costs to the patient |  |  |  |  |  |
| 1. When choosing treatment, doctors should consider costs to society (i.e. how treatment of individual patients affects the health care system) |  |  |  |  |  |
| 1. Patients should have access to the costs of their treatment before making treatment decisions |  |  |  |  |  |
| 1. I feel prepared to discuss costs of treatment with patients |  |  |  |  |  |
| 1. I feel comfortable discussing costs of treatment with patients |  |  |  |  |  |
| 1. I have easy access to quality resources that assist me in cost discussions with patients |  |  |  |  |  |
| 1. My consideration of health care costs varies based on my patient’s insurance status or socioeconomic background. |  |  |  |  |  |
| 1. If two treatments are equally effective, I believe doctors should recommend the less expensive option |  |  |  |  |  |
