# Supplementary material for: Implementation of an educational intervention to improve medical student cost awareness: a prospective cohort study
Source: BMC Med Educ. 2023 Jan 30;23:73. doi: 10.1186/s12909-023-04038-1 (PMC9885673; doi:10.1186/s12909-023-04038-1)
Supplement: Supplementary file 4 — Additional file 4. Intervention Cohort Two Month Post-Session Follow-Up Survey [file 12909_2023_4038_MOESM4_ESM.docx]

**Additional File 4.** Intervention Cohort Two Month Post-Session Follow-Up Survey

1. To maintain anonymity, please follow the instructions to create an ID that will be used to match your pre- and post-participation surveys.

-Initial of first name

-Last letter of last name

-First letter of birth month

-First letter of mother’s maiden name

Example: Susan Smith born in June, mother’s maiden name Jones: SHJJ

1. In your experience, how often do patients consider their personal out-of-pocket or indirect costs when making treatment decisions?
2. All of the time
3. Most of the time
4. Sometimes
5. Infrequently
6. Never
7. How often do you think about patient out-of-pocket costs (i.e. deductibles and co-pays) when considering treatment options?
8. All of the time
9. Most of the time
10. Sometimes
11. Infrequently
12. Never
13. How often have you used the pocket tool to assist in cost discussions with patients?
    1. 10+ times
    2. 5-10 times
    3. 1-5 times
    4. Never
14. How often have you engaged in cost discussions with patients over the past 2 months?
15. All of the time
16. Most of the time
17. Sometimes
18. Infrequently
19. Never
20. Which of the following are barriers for you to discussing costs of treatment with patients? (select all that apply)
21. I don’t know enough about the costs of care/lack resources
22. Not enough time to discuss costs
23. I can’t help with the costs of care
24. It’s not my place to discuss costs of care
25. It is uncomfortable to discuss costs with patients
26. Discussing costs might impact the quality of care patients receive
27. Nothing prevents me from discussing costs
28. Other
29. How often have you engaged in cost discussions with the rest of the medical team over the past 2 months?
30. All of the time
31. Most of the time
32. Sometimes
33. Infrequently
34. Never
35. Which of the following are barriers for you to discussing costs of treatment with the rest of the medical team? (select all that apply)
36. I don’t know enough about the costs of care/lack resources
37. Not enough time to discuss costs
38. I can’t help with the costs of care
39. It’s not my place to discuss costs of care
40. It is uncomfortable to discuss costs with the medical team
41. Discussing costs might impact the quality of care patients receive
42. Nothing prevents me from discussing costs
43. Other
44. Please share any additional comments you have regarding your personal thoughts, experiences or beliefs on financial toxicity.
45. The following statements are aimed at helping us understand how health care providers think about treatment costs- including costs to patients and the health care system. Please indicate how much you agree or disagree.

|  | **Strongly**  **Agree** | **Agree** | **Neither Agree nor Disagree** | **Disagree** | **Strongly Disagree** |
| --- | --- | --- | --- | --- | --- |
| 1. I have a good understanding of the following terms: deductibles, co-payment, co-insurance, maximum out of pocket cost |  |  |  |  |  |
| 1. Doctors should explain to patients the costs the patient will have to pay for his or her treatment |  |  |  |  |  |
| 1. When choosing treatment, doctors should consider costs to the patient |  |  |  |  |  |
| 1. When choosing treatment, doctors should consider costs to society (i.e. how treatment of individual patients affects the health care system) |  |  |  |  |  |
| 1. Patients should have access to the costs of their treatment before making treatment decisions |  |  |  |  |  |
| 1. I feel prepared to discuss costs of treatment with patients |  |  |  |  |  |
| 1. I feel comfortable discussing costs of treatment with patients |  |  |  |  |  |
| 1. I have easy access to quality resources that assist me in cost discussions with patients |  |  |  |  |  |
| 1. My consideration of health care costs varies based on my patient’s insurance status or socioeconomic background. |  |  |  |  |  |
| 1. If two treatments are equally effective, I believe doctors should recommend the less expensive option |  |  |  |  |  |
